# Brain oxidative stress mediates anxiety-like behavior induced by indomethacin in zebrafish: protective effect of alpha-tocopherol

**DOI:** 10.1007/s00210-023-02661-9

**Published:** 2023-09-18

**Authors:** Jessica Pinheiro, Emerson Pinheiro, Gustavo Ramalho de Deus, Geovanna Saito, Waldo Lucas Luz, Nadyme Assad, Melk Roberto da Cunha Palheta, Evander de Jesus Oliveira Batista, Suellen Morais, Adelaide Passos, Karen Renata Herculano Matos Oliveira, Anderson Manoel Herculano

**Affiliations:** 1 Laboratory of Experimental Neuropharmacology, Institute of Biological Sciences, Federal University of Pará, Belém, Brazil; 2 Laboratory of Protozoology, Tropical Medicine Center, Federal University of Pará, Belém, Brazil

**Keywords:** NSAIDs, Indomethacin, Anxiety-like behavior, Oxidative stress, Zebrafish

## Abstract

**Rationale:**

Indomethacin (INDO) is a widely utilized non-steroidal anti-inflammatory drug (NSAID) with recognized effect on the central nervous system. Although previous reports demonstrate that prolonged treatment with indomethacin can lead to behavioral alterations such as anxiety disorder, the biochemical effect exerted by this drug on the brain are not fully understood.

**Objectives:**

The aim of present study was to evaluate if anxiety-like behavior elicited by indomethacin is mediated by brains oxidative stress as well as if alpha-tocopherol, a potent antioxidant, is able to prevent the behavioral and biochemical alterations induced by indomethacin treatment.

**Methods:**

*Z*ebrafish were utilized as experimental model and subdivided into control, INDO 1 mg/Kg, INDO 2 mg/Kg, INDO 3 g/Kg, α-TP 2 mg/Kg, α-TP 2 mg/Kg + INDO 1 mg/Kg and α-TP + INDO 2 mg/Kg groups. Vertical distributions elicited by novelty and brain oxidative stress were utilized to determinate behavioral and biochemical alterations elicited by indomethacin treatment, respectively.

**Results:**

Our results showed that treatment with indomethacin 3 mg/kg induces animal death. No changes in animal survival were observed in animals treated with lower doses of indomethacin. Indomethacin induced significant anxiogenic-like behavior as well as intense oxidative stress in zebrafish brain. Treatment with alpha-tocopherol was able to prevent anxiety-like behavior and brain oxidative stress induced by indomethacin.

**Conclusions:**

Data presented in current study demonstrated for the first time that indomethacin induces anxiety-like behavior mediated by brain oxidative stress in zebrafish as well as that pre-treatment with alpha-tocopherol is able to prevent these collateral effects.

## Introduction

Indomethacin (INDO) is a non-steroidal anti-inflammatory drug (NSAID) with analgesic, antipyretic, and anti-inflammatory properties (Hunskaar and Hole 1987; Lucas 2016; Panchal and Prince Sabina 2023). Previous studies have shown that indomethacin is utilized for treating important diseases such as osteoarthritis and rheumatoid arthritis (Crofford 2013; Graham 1993). Although this drug consists of a widely consumed medicine, a considerable number of reports describe significant collateral effects associated with prolonged treatment with indomethacin (El-Mashad et al. 2017; Lövgren and Allander 1964; Sawdy et al. 2003; Seideman and Arbin 1991; Yeh et al. 1982). The literature reports that abdominal pain, heartburn and diarrhea are the main side effects caused by the excessive use of NSAIDs. These effects result from the blockade of COX-1, which results in a reduction in the synthesis of prostaglandins and prostacyclin (PGE2 and PGD2) in the gastrointestinal mucosa. Furthermore, studies report that the hepatic system is also affected by the indiscriminate use of NSAIDs. Damage to hepatic tissue can be observed in two stages: The first is acute hepatitis, which is characterized by jaundice, fever, nausea, elevated transaminases and eosinophilia. The second stage is characterized by periportal inflammation, plasma and lymphocyte infiltration, culminating in active chronic hepatitis (Bessone 2010; Haag 2008; Lanza et al. 1979; Panchal and Prince Sabina 2023).

Previous studies demonstrate that the indiscriminate use of NSAIDs causes severe damage to the central nervous system. The main neuronal symptoms related to its use are drowsiness, confusion, blurred vision, diplopia, headache, various types of strokes and, consequently, cognitive damage, spatial memory and recognition deficits (McCulloch et al. 1982). Past reports demonstrate important cognitive impairment in patients treated with INDO (Clark and Ghose 1992; Hoppmann et al. 1991). In addition, previous articles also demonstrate that NSAIDs evoke severe brain dysfunction and intense behavior alterations such as anxiety in patients (Morgan and Clark 1998; Onder et al. 2004). In face of these effects, studies aimed to describe the neural mechanisms associated with brain toxicity elicited by INDO can be very useful to prevent the behavioral changes elicited by this drug.

It is well documented in the literature that the main effect of INDO on biological systems is the inhibition of cyclooxygenases (COX), particularly cyclooxygenase-2 (COX-2), which represents an important enzyme for controlling inflammatory response (Chauhan et al. 2018; Chan et al. 2016; Rouzer and Marnett 2009; Seibert et al. 1994). Although few studies have described the action mechanism of indomethacin on the brain, there are strong pieces of evidence demonstrating that treatment with indomethacin induces changes in neurotransmitter systems. Although few studies have described the action mechanism of indomethacin on the brain, there are strong pieces of evidence demonstrating that treatment with indomethacin induces changes in neurotransmitter systems. This drug use can reduce synaptic vesicle fusion events of the glutamatergic system, caused by activation of both purinergic and glutamatergic receptors. Furthermore, the indomethacin can induce the indirect activation of acetylcholine receptors and consequently the increase of glutamate release (Cali et al. 2014; Kanno et al. 2012; Phillis et al. 1994; Pitcher and Henry 1999; Li et al. 2009). Previous reports also showed that prolonged treatment with INDO can evoke oxidative stress in different biological systems like the in liver, kidney and brain tissues, and this phenomenon is associated with decreased activity of Glutathione (GSH), Superoxide dismutase (SOD) and Catalase (CAT) (Ahmad and Mondal 2018; Handa et al. 2014; Hegab et al. 2018; Khan et al. 2019).

Data from literature reports that oxidative stress acts as a molecular inductor of changes in the tissues homeostasis by altering macromolecules structures such as lipid membranes, DNA and proteins (Balmus et al. 2016; Hovatta et al. 2010; Lv et al. 2019; Malcon et al. 2020). According Fedoce et al. (2018) when this imbalance occurs in brain tissue it can leads to significant behavioral alterations such as panic disorder, depression and anxiety disorder. In this way, the current study aimed to evaluate the participation of oxidative stress as a mechanism of indomethacin-induced anxiety by utilization of a potent antioxidant. Alpha-tocopherol (α-TP) is one of the different isoforms of vitamin E and represents one of the most efficient antioxidants in biological systems (Kamal-Eldin and Appelqvist 1996; Na et al. 2021; Wallert et al. 2019). It is well documented that α-TP can easily cross the blood–brain barrier, having action on the CNS (Lee and Ulatowski 2019; Rigotti 2007). As previously demonstrated by our group, α-TP blocked the brain effects generated by high caffeine consumption even after systemic administration in zebrafish (De Carvalho et al. 2019).

Anxiety-like behavior is an evolutionarily conserved behavior observed in different species, including mammals and fish (Ausderau et al. 2023; Lang et al. 2000; Kysil et al. 2017). Several studies have demonstrated that *Danio rerio* (zebrafish) represents a powerful model to evaluate mechanisms controlling altered behavior such as anxiety (Assad et al. 2020; Kalueff et al. 2014; Maximino et al. 2011). In fact, it is well documented that molecules inducing anxiogenic effects on humans exert a similar effect on zebrafish (López-Patiño et al. 2008; Mathur and Guo 2011; Stewart et al. 2011). This species also presents brain regions analogous to those involved in the controlling of anxiety-like behavior in humans, as well as classical vertebrate neurotransmitters such as glutamate, GABA, and serotonin (Assad et al. 2020; Cognato et al. 2012; Kaslin and Panula 2001; Maximino et al. 2013a, b; Maximino et al. 2013a, b). In addition, several toxicological studies have already used the zebrafish to evaluate effect of drugs on redox homeostasis, suggesting this animal as promising model for the field of drug discovery that modulation oxidative stress (Mugoni et al. 2014). All these characteristics make these animals an excellent experimental model for studies aiming to describe neural events controlling altered behavior such as anxiety disorder. Therefore, the present study aims to evaluate the neuroprotective role of α-TP treatment against the behavioral and biochemical effects generated by the systemic administration of indomethacin in zebrafish.

## Material and methods

### Animals and housing

Eighty-six *Danio rerio* (zebrafish) *long fin* from 3–4 months old, weighing 0.4 g (± 0.2) from both sexes (50:50 ratio), were purchased from a local supplier (Belém-Pará). Fish were acclimated in 50 L tanks (50 × 35 × 30) at 25 ºC ± 2, pH 6.5, oxygenation, 14-h/10-h light/dark controlled photoperiod and fed once a day with commercial flocculated feed (Tetra, Germany) with density of 1 animal per liter and were acclimatized for a minimum period of 15 days, according to previous studies performed by our group. All experimental procedures were made in accordance with the National Council of Animal Experimentation Control (CONCEA) and previously approved by the Committee of Ethics in Research with Experimental Animals of the Federal University of Pará (CEPAE—UFPA: 213–14).

### Drug administration

**Fig. 1:**
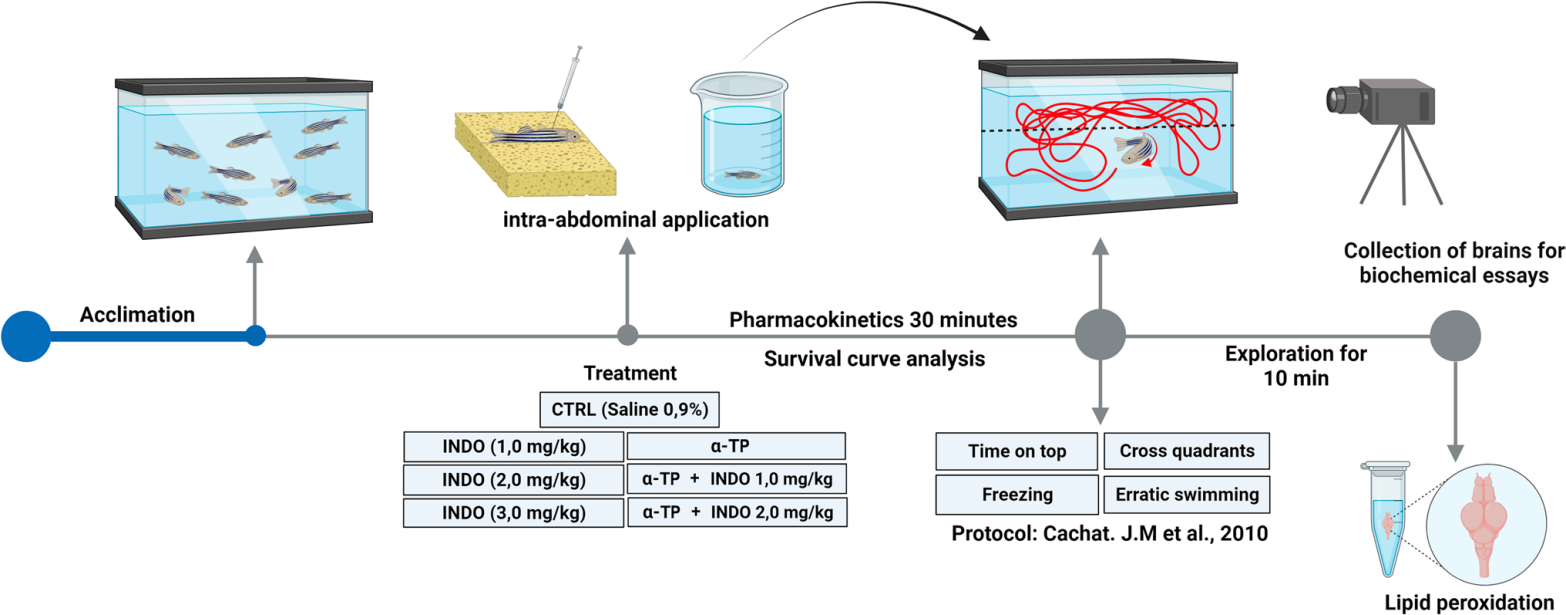
Timeline of experiments, starting with acclimatization, pharmacological applications followed by behavioral tests and brain collection for biochemical assays. (Illustration produced using BioRender.com, free version)

Drugs used in the current study were: Indomethacin (INDO) at concentrations of 1, 2 and 3 mg/kg; alpha-tocopherol (α-TP) at a concentration of 2 mg/kg, all diluted in 1% Dimethylsulfoxide (DMSO) (Hoyberghs et al. 2021). For the biochemical assay, were used N-methyl-2 phenylindole (NMFI), fetal bovine serum (FBS), methanesulfonic acid, malondialdehyde (MDA) all were purchased from SIGMA-ALRICH Company. For drug administration, the animals were individually cryoanesthetized in cold water at 2ºC followed by the application intra-abdominally (i.a) using Hamilton® syringe as previously described by Assad et al (2020). After the procedure, the animals were relocated separately in acclimatization aquarium for 30 min before the tests (Fig. 1).

### Experimental design

A dose–response curve was performed to determine indomethacin toxicity the first 30 min after the drug injection, this evaluation was carried out by intra-abdominally (i.a) injection of 5 µl indomethacin at 1 mg/kg, 2 mg/kg and 3 mg/kg in zebrafish (n = 8 per group). The dose of indomethacin which evoked a decrease in the zebrafish survival rate was excluded from posterior experiments. Control and experimental groups were classified as follows: Control (CTRL—0.9% saline solution), Indomethacin (INDO—1 mg/kg, 2 mg/kg or 3 mg/kg), alpha-tocopherol (α-TP—2 mg/kg) and α-TP (2 mg/kg) + INDO (1 mg/kg or 2 mg/kg) (n = 8–10 per group). All behavioral tests were performed between 8:00 AM and 1:00 PM.

### Novel-tank diving test (Geotaxis)

Animals were tested in the novel-tank diving test as previously described by Egan et al. (2009) and Cachat et al. (2010). Briefly, 30 min after injections of vehicle or drugs, zebrafish were individually transferred to behavioral apparatus which consisted of a glass aquarium (15 cm x 25 cm x 20 cm, width x length x height). The free exploration of the animal in the apparatus was recorded with a digital camera for 10 min, we have considered as the top zone the upper region of apparatus which consisted in 10 cm region measured from the middle of aquarium to the top of water column. The following variables were recorded: time on top (s): the time spent in the top third of the tank; number squares crossed (n): the values of squares crossed were determinate as the number of 5 cm^2^ squares crossed by the animal during the entire session; erratic swimming (n): the number of “erratic swimming” events, defined as a zig-zag, fast, unpredictable course of swimming of short duration (< 3 s); and freezing (s): the total duration of freezing events, defined as complete cessation of movements with the exception of eye and operculae movements, with the minimum duration of 5 s (Maximino et al. 2013a, b). Videos analyses were performed by double-blind evaluation using the software X-Plo-Rat 2005.

### Biochemical assay

Brain oxidative stress in control and treated groups was measured by determining the malondialdehyde (MDA). This method consists in the quantification of molecular products caused by oxidative stress, allowing to indirectly infer the levels of lipid peroxidation in a tissue, using the colorimetric method described by Gérard-Monnier et al. (1998). After the cryoanesthesia, animals were quickly decapitated, and their brain tissue was dissected and stored in micro-tube containing 300 µL of TRIS–HCl buffer (pH 7.4) at -80ºC until the time of analysis. This step was followed by tissue sonication and homogenization as previously described by Pinheiro et al. (2022). The homogenate was then centrifuged at 5600 rpm at 4 °C for 10 min. MDA levels in the samples were determined by reaction at 37 °C in the presence of 10 mM N-methyl-2 phenylindole (NMFI) and methanesulfonic acid solution. Lipid peroxidation in the brains was analyzed based on standard curve concentrations of malondialdehyde (MDA), measured by absorbance at λ = 570 nm. MDA concentration was quantified in nmol per milligram of protein, and protein levels were determined by the Bradford method. The values were expressed as a percentage of the control.

### Statistical analysis

Data are presented as mean and standard of mean error (S.E.M) for behavioral analysis and percentage of controls for biochemical analysis. The normal distribution of data was determined by the Shapiro–Wilk test. One-way analysis of variance (ANOVA) followed by Tukey's post hoc test was applied to evaluate the biochemical and behavioral data. Log-rank Kaplan–Meier curve test was utilized to analyze the survival curve. All analyzes were performed using the GraphPad Prism software version 9.3.0 (GraphPad Software Inc., San Diego, CA, USA), with a significance level of p < 0.05.

## Results

### Indomethacin toxicity in zebrafish

**Fig. 2:**
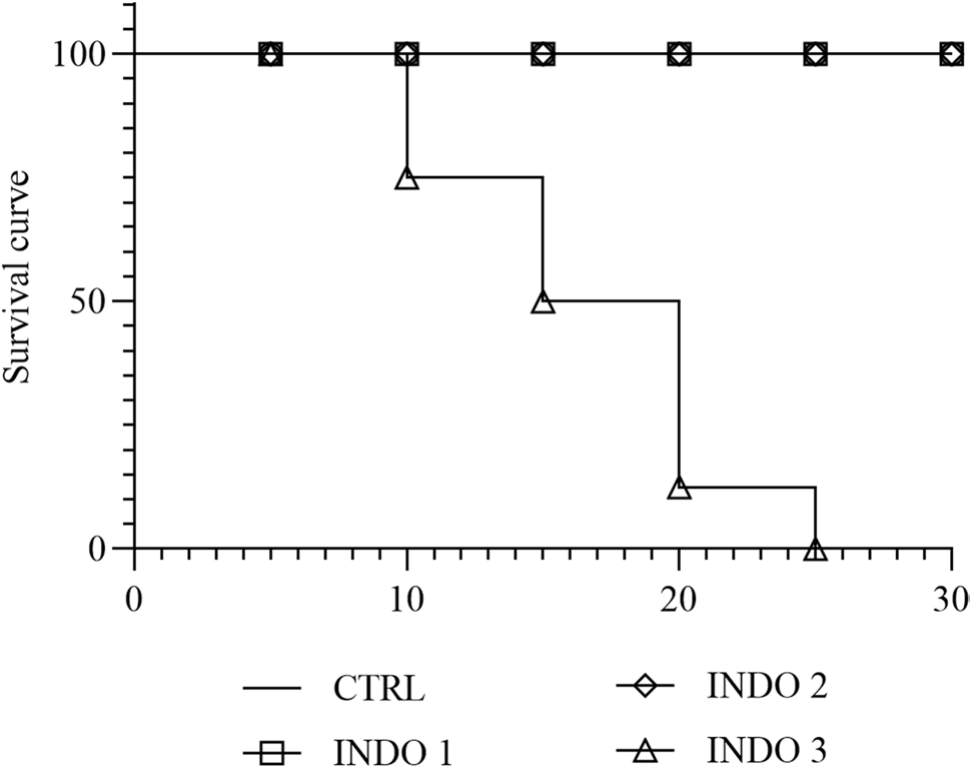
Effect of indomethacin on zebrafish survive. Control Saline 0,9% = CTRL, Indomethacin 1 mg/Kg = INDO 1, Indomethacin 2 mg/Kg = INDO 2 and Indomethacin 3 mg/Kg = INDO 3. Data are expressed as mean ± SD. n = 8 animals/group

To address the effect of indomethacin toxicity at different doses, subjects received INDO 1 mg/kg, 2 mg/kg, and 3 mg/kg and CTRL (0.9% NaCl) intra-abdominally and were observed for 30 min. Survival data showed that treatment with indomethacin at 3 mg/kg promoted significant toxicity, inducing 100% of mortality after 10–25 min (Fig. 2). 1 mg/kg and 2 mg/kg of indomethacin did not exert alterations in the animal survive as observed in Fig. 2.

### Indomethacin treatment induces anxiety-like behavior

**Fig. 3:**
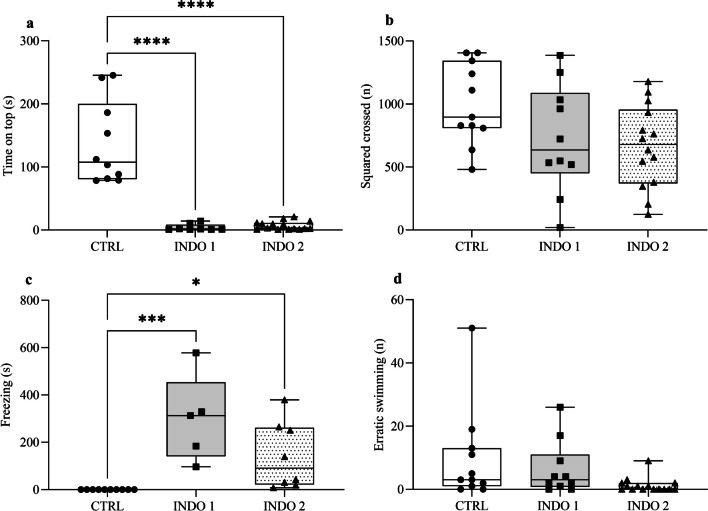
Effect of indomethacin on (**a**) time on top, (**b**) squares crossed, (**c**) Freezing and (**d**) erratic swimming test. Control Saline 0,9% = CTRL (n = 10), Indomethacin 1 mg/Kg = INDO 1 (n = 8) and Indomethacin 2 mg/Kg = INDO 2 (n = 15). Graphs represent the mean ± S.E.M and comparisons were made using ANOVA one-way test followed by the Tukey test. ****p < 0,00001 vs. CTRL, ***p < 0,0001 vs. CTRL, *p < 0,001 vs. CTRL

Results of the novel tank diving test demonstrated that indomethacin induces anxiety-like behavior in zebrafish. The animals treated with indomethacin at 1 mg/kg and 2 mg/kg spent less time at the top of the apparatus (Fig. 3a: *F*_(2, 30)_ = 44,61; CTRL = 136.8 ± 20.90 *vs.* INDO 1 = 3.98 ± 1.88 vs. INDO 2 = 7.06 ± 1.72; p < 0.0001). This data demonstrate that indomethacin induces more than 80% reduction in time spent in the top of apparatus suggesting a potent anxiogenic effect. Data of squares crossed evaluation showed that indomethacin had no significant effect on the locomotion of treated zebrafish when compared with control group (Fig. 3b). However, we also evidenced that animals treated with indomethacin at 1 mg/kg and 2 mg/kg had an increase freezing time compared to the control group (Fig. 3c: *F*_(2, 20)_ = 11,35; CTRL = 0.06 ± 0.04 vs. INDO 1 = 300.20 ± 81.66 vs. INDO 2 = 141.70 ± 49.81; p = 0,0005). The erratic swimming values did not show significant differences between the groups treated with indomethacin and the control group (Fig. 3d).

### The oxidative stress induced by Indomethacin in zebrafish brain is prevented by the alpha-tocopherol

**Fig. 4:**
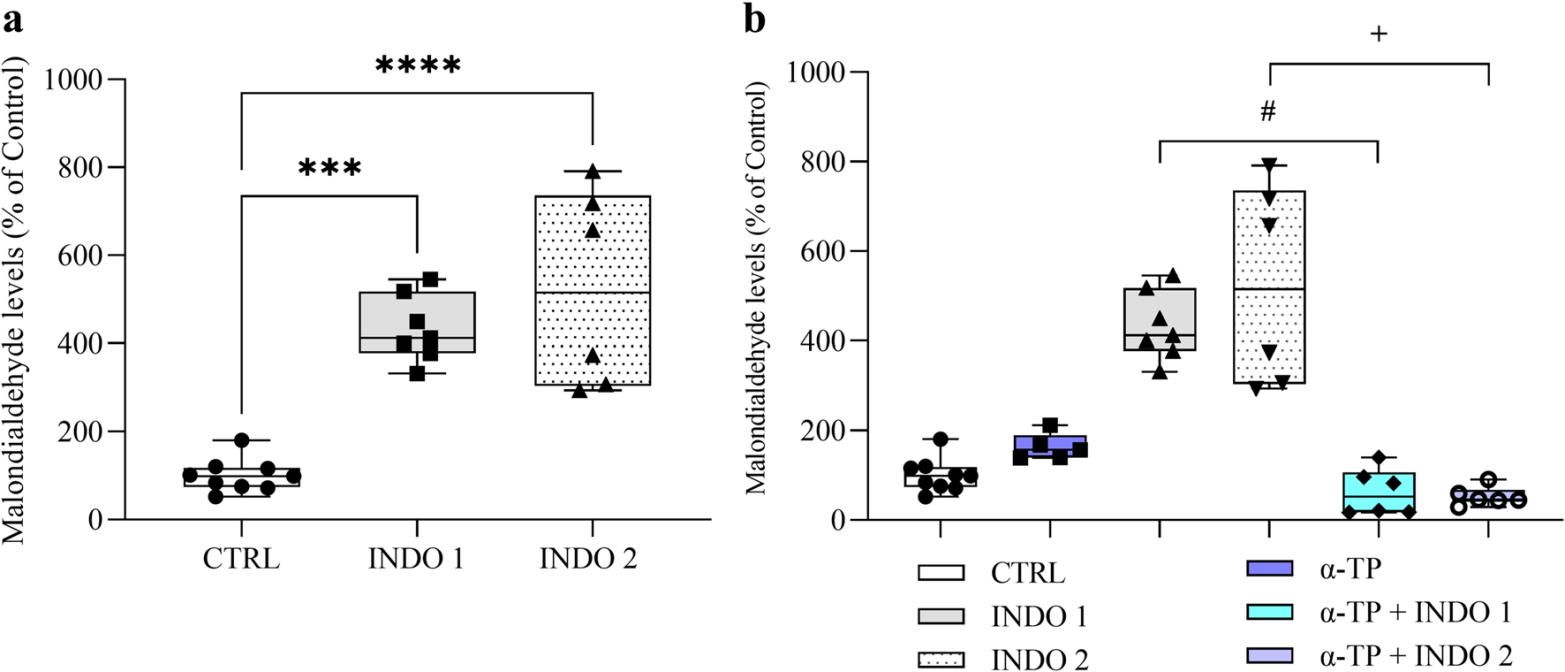
Lipid peroxidation in zebrafish brain (**a**, **b**). Control Saline 0,9% = CTRL (n = 9), Indomethacin 1mg/Kg = INDO 1 (n = 7) and Indomethacin 2mg/Kg = INDO 2 (n = 6). Data are expressed as percent of control ± S.E.M. Data were compared using ANOVA-one way test followed by the Tukey test. ****p < 0,00001 vs. CTRL, ***p < 0,0001 vs. CTRL, ^#^p < 0,00001 vs. INDO 1, ^+^p < 0,0001 vs. INDO 2

Indomethacin treatment induced an increase in MDA levels in the zebrafish brain after 30 min of exposure (Fig. 4a: *F*_(2, 19)_ = 24,94; CTRL = 100.00 ± 12.43 vs. INDO 1 = 433.61 ± 28.83 vs. INDO 2 = 523.50 ± 91.10; p < 0,0001). As observed in Fig. 4, treatment with α-TP prevented MDA elevation induced by indomethacin at doses 1 mg/kg and 2 mg/kg in the zebrafish brain (Fig. 4b: F _(5, 33)_ = 28,07; α-TP + INDO 1 = 62.42 ± 21.04 vs. INDO 1 = 433.61 ± 28.83; p = 0.0003 and α-TP + INDO 2 = 52.33 ± 8.58 vs. INDO 2 = 523.50 ± 91.10; p < 0,0001). α-TP treatment did not have a significant effect on MDA levels compared to the control group, as well as the groups pretreated with α-TP.

### Alpha-tocopherol prevents indomethacin-induced anxiety-like behavior

**Fig. 5:**
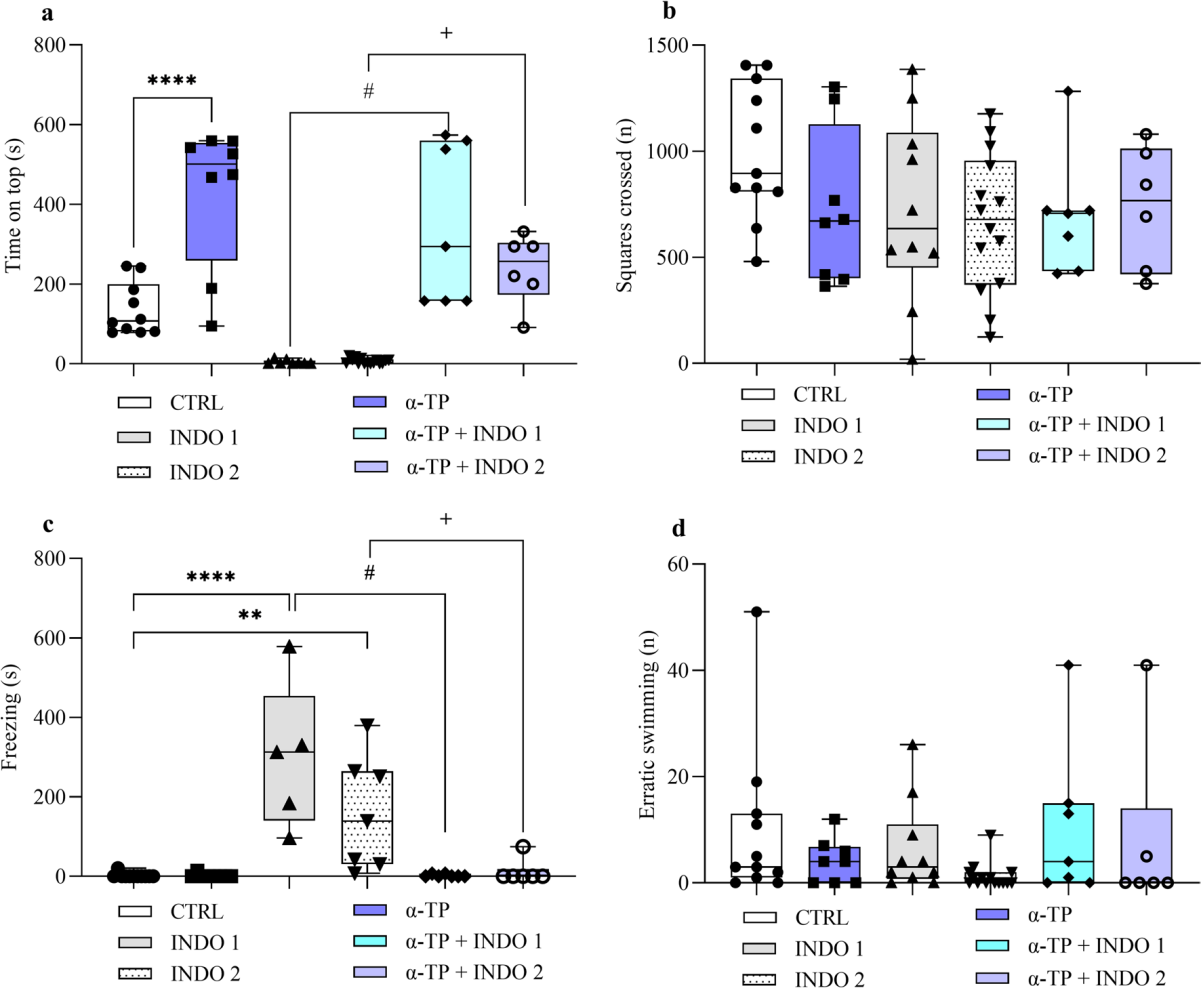
Effect of alpha-tocopherol on (**a**) time on top, (**b**) squares crossed, (**c**) Freezing and (**d**) erratic swimming in the geotaxy of zebrafish treated with indomethacin. Control Saline 0,9% = CTRL (n = 10), Indomethacin 1 mg/Kg = INDO 1 (n = 8), Indomethacin 2 mg/Kg = INDO 2 (n = 15), Alpha-Tocopherol = α-TP (n = 8), α-TP + INDO 1 (n = 7) and α-TP + INDO 2 (n = 6). Graphs represent the mean ± S.E.M. Data were compared using ANOVA-one way test followed by the Tukey test. ****p < 0,00001 vs. CTRL, **p < 0,001 vs. CTRL, ^#^p < 0,0001 vs. INDO 1, ^**+**^p < 0,0001 vs. INDO 2

Our data showed that alpha-tocopherol pre-treatment prevented the indomethacin-induced 1 mg/kg and 2 mg/kg decrease in exploration time on top: (Fig. 5a: F _(5, 48)_ = 24,48; α-TP + INDO 1 = 338.79 ± 74.76 vs. INDO 1 = 3.98 ± 1.75; p < 0.0001 and α-TP + INDO 2 = 201.87 ± 38.87 vs. INDO 2 = 7.06 ± 1.72; p < 0,0001). In addition, we observed that pre-treatment with α-TP promoted a potent anxiolytic effect, with an increased exploration time at the top compared to the control group, both for individuals who received indomethacin or not: (Fig. 5a: F _(5, 48)_ = 24,48; CTRL = 129.73 ± 19.25 vs. α-TP = 426,8 ± 63,91 vs. α-TP + INDO 1 = 338.79 ± 74.76 vs. α-TP + INDO 2 = 201.87 ± 38.8; p < 0,0001). Alpha-tocopherol also blocked indomethacin-induced freezing behavior at both doses: (Fig. 5c: F _(5, 38)_ = 13,39; α-TP + INDO 1 = 1.94 ± 1.25 vs. INDO 1 = 300 ± 181.66; p < 0.0001 and α-TP + INDO 2 = 12.43 ± 12.43 vs. INDO 2 = 159.20 ± 53.82; p = 0,0227). However, there were no significant differences between the groups treated with α-TP and α-TP + INDO with the control (Fig. 5c). Analysis of the number of squares crossed (Fig. 5b) and erratic swimming (Fig. 5d) no showed a significant among the groups.

## Discussion

Data presented in the current study demonstrated for the first time that brain oxidative stress mediates indomethacin-induced anxiety-like behavior. These behavioral and oxidative changes were prevented by treatment with α-TP, which is a potent antioxidant with neuroprotective action against damage caused by redox desbalance. Our data are in agreement with previous studies demonstrating neuropsychological dysfunctions associated to adverse effect elicited by treatment with non-steroidal anti-inflammatory drugs (NSAIDs) (Adhikary et al. 2011; Bercik et al. 2010; Goodwin et al. 2013). In fact, animal models have emerged as an important tool for elucidating the toxicological events associated with indomethacin’s action in the brain (Benesová et al. 2001; Enos et al. 2013). Anterior studies already demonstrated that indomethacin amplifies the anxiety-like behavior in animals submitted to physical stress (Fernández-Guasti and Martínez-Mota 2003). Our study demonstrated that zebrafish displayed similar anxiogenic-like behavior when treated with different doses of indomethacin. Subjects treated with indomethacin spent less time in the upper portion of the apparatus when compared with controls. Data demonstrating that indomethacin treatment does not exert significant locomotor alterations, ratify our interpretation that indomethacin evoked anxiogenic behavior in zebrafish. In other words, the time spent by the subjects at the bottom of the apparatus is provoked by anxiety-like behavior, but not by motor impairment elicited by indomethacin treatment (Neelkantan et al. 2013; Rosemberg et al. 2012). This behavior is also observed in subjects submitted to classical anxiogenic compounds such as alarm substance (Lima-Maximino et al. 2020), sub-convulsant doses of pentylenetetrazol (Wong et al. 2010) or caffeine (De Carvalho et al. 2019). In this way, our finds ratify the literature demonstrating that zebrafish represent a powerful animal model to evaluate neurochemical and behavioral changes associated with drugs leading to a collateral effect on the central nervous system (Maximino et al. 2014; Mocelin et al. 2015; Li et al. 2015).

It has been described in the literature that indomethacin can modulate inflammation and oxidative stress in the CNS (Adhikary et al. 2011; Bercik et al. 2010) which may cause altered behavior such as anxiety (Hassan et al. 2014; Masood et al. 2008; Zhang et al. 2019; Zheng et al. 2021). Our data have supported this hypothesis since animals treated with indomethacin showed intense oxidative stress in their brain. Although some studies point out that treatment with NSAIDs induces a decrease in anti-inflammatory cytokines, there is strong evidence demonstrating that long treatment with indomethacin can promote the overproduction of reactive oxygen species and oxidative stress in different tissues (Ahmad and Mondal 2018; Farooq et al. 2007; Khan et al. 2019). We demonstrated in the present study the close relation between indomethacin-induced anxiety and oxidative stress in the zebrafish brain since treatment with alpha-tocopherol, a potent antioxidant, was able to prevent both anxiety-like behavior and oxidative stress in the brain of animals treated with indomethacin. Our data are aligned with previous studies describing that treatment with antioxidants such as vitamin C (Puty et al. 2014) and alpha-tocopherol (De Carvalho et al. 2019) can prevent anxiety-like behavior in zebrafish. In rodent models, several studies also have reported the involvement of oxidative stress in anxiety-like behaviors (Dhingra et al. 2014; Vollert et al. 2011). Desrumaux et al. (2018) demonstrated that decreased levels of alpha-tocopherol and increased levels of central oxidative stress markers, such as cholesterol oxides and cellular peroxides, result in anxiety-like behavior in mice. In addition, the increase in lipid peroxidation alters the levels of antioxidant defenses, such as glutathione, in addition to generating DNA damage and reducing the activity of antioxidant enzymes (Bouayed et al. 2009; Ferreira Mello et al. 2013; Jangra et al. 2014).

## Conclusion

Indomethacin induces anxiety-like behavior and lipid peroxidation in brain tissue of zebrafish. Protective effect exerted by alpha-tocopherol treatment against indomethacin-induced behavioral and biochemical alterations let us to conclude that indomethacin evokes anxiety by generation of oxidative stress in zebrafish brain. These findings strongly suggest that generation of oxidative stress represents an important mechanism of generation of anxiety elicited by indomethacin treatment. Our data also support that utilization of antioxidants could be an efficient strategy to prevent the deleterious effects of indomethacin on the CNS.

## Data Availability

Not applicable.
